# World TB Day 2018: The Challenge of Drug Resistant Tuberculosis

**DOI:** 10.12688/f1000research.14088.1

**Published:** 2018-02-22

**Authors:** Ankur Gupta-Wright, Gillian S Tomlinson, Molebogeng X Rangaka, Helen A Fletcher

**Affiliations:** 1 TB Centre, London School of Hygiene & Tropical Medicine, London, UK; 2 Infection & Immunity , University College London, London, UK; 3 Institute for Global Health , University College London, London, UK; 4 School of Public Health , University of Cape Town, Cape Town, South Africa

**Keywords:** Drug resistant TB, tuberculosis

## Abstract

On 24th March, the world commemorates the day in 1882 when Dr Robert Koch announced his discovery of *Mycobacterium tuberculosis* (MTB). Over 130 years later, tuberculosis (TB) continues to affect individuals, communities, and entire health systems and economies. Koch unsuccessfully tried to ‘cure’ TB, and despite major advances in other areas of medicine, control of TB remains elusive- in 2016 TB was the leading infectious cause of death. The STOP TB partnership and World Health Organization (WHO) have announced their theme for World TB Day 2018 “Wanted: Leaders for a TB-Free World. You can make history. End TB.” This theme recognizes that TB is much larger than any one person, institute or discipline of research, and provides an opportunity for us to reflect on the major challenges and consider how we, as a scientific community, can work together and take the lead to address the global crisis of drug-resistant TB (DR-TB).

**Editorial:**

TB was declared a global emergency in 1993 and great efforts have been made to improve cure rates and reduce mortality from drug-sensitive tuberculosis. However, the widespread emergence of DR-TB threatens to overthrow decades of progress. TB is the most widespread airborne anti-microbial resistant (AMR) disease worldwide and, in September 2017, the WHO declared TB a priority pathogen for the development of new antibiotics. The pressure of coping with drug resistant TB on patients, health systems and economies is magnitudes of scale greater than that of drug sensitive TB, which is itself already a huge burden. The problem has been amplified by decades of under investment in TB research and development which has left us with knowledge gaps and limited tools to deal with this new threat.

Questions we would like the scientific community to reflect on for World TB Day 2018 include:

1) What is drug resistant TB, where is it and how is it spread?

2) What are we doing about drug-resistant TB?

3) What new approaches should we consider?

## What is drug resistant TB, where is it and how is it spread?

Resistance to key first-line anti-TB drugs manifests most commonly as rifampicin-resistant and multi-drug resistant (MDR) TB, and continues to increase despite overall global reductions in TB incidence. The gap between notified cases ( ^~^150,000 globally in 2016) and estimated burden ( ^~^600,000 globally) also widens ^1^. More concerningly, global estimates indicate that only 1 in 10 patients with MDR-TB are successfully treated. It is not coincidence that the highest drug-resistant TB (DR-TB) burden concentrates in the parts of Europe, Africa and Asia with the weakest health systems, and reflects failures at all stages of the DR-TB care pathway, including seeking care, diagnosis of TB and drug-resistance, and supporting a long, and often toxic treatment regimen.

Furthermore, we are only recently beginning to understand the impact that failing to control DR-TB has on onward transmission, with potentially devastating consequences. Whereas strengthening the delivery of drug-sensitive TB care was a priority for managing DR-TB through prevention of *de novo* resistance, other strategies will be required to deal with the ongoing transmission of DR-TB. There are still too many unknowns in our understanding of how much and where DR-TB occurs, and how it spreads.

## What are we doing about drug resistant TB?

This is not to say there hasn’t been progress in managing DR-TB. Traditionally, diagnosis of DR-TB required culture and drug-susceptibility testing, with very long turnaround times, and lack of facilities and expertise where the need was greatest, contributing to under-diagnosis and pre-treatment loss-to-care. However, the development and scaling-up of near patient molecular testing technologies, for example the Xpert MTB/RIF assay (Cepheid, USA), has already been utilized to accurately detect rifampicin-resistance, with promising progress in detecting second-line drug (mainly fluoroquinolones and aminoglycosides) susceptibility using similar technologies.

Existing treatments for DR-TB are lengthy, require patients to take tens of pills and attend health facilities for injections, have modest efficacy and commonly cause debilitating side-effects. The first new anti-TB drugs for over 40 years – delamanid and bedaquiline – have been reserved for DR-TB. Data emerging from observational and randomized studies suggests good outcomes can be achieved with shorter regimens and fewer drugs. Combining these with improved diagnostics offers some hope of improving successful treatment completion rates.

Molecular techniques and whole-genome sequencing have also improved understanding of DR-TB transmission, and genomic evidence strongly supports transmission of DR strains in multiple settings. Yet the evidence on how to manage contacts of DR-TB who have potentially been exposed is still lacking, especially those at highest risk, for example children or people living with HIV. Randomized trials of preventive drug therapies are ongoing. We have also begun to describe the catastrophic costs and impact of DR-TB on individuals, families, communities and health systems, as, even highly efficacious interventions cannot be delivered successfully without addressing these ^2^.

## What new approaches should we consider?

Our understanding of the TB bacillus has progressed since Koch’s discovery. Despite MTB’s genome being first sequenced in 1998, many questions remain unanswered about both the pathogen and host-pathogen interactions. Although it is clear that MTB utilizes and manipulates human host responses to facilitate its survival and onward transmission, our understanding of how to influence these interactions for prevention of infection and/or disease is incomplete. 

Identifying the immunological correlates of protection and pathogenesis is a key priority in TB research. This knowledge is crucial to inform development of host directed therapies which may improve outcomes and shorten treatment duration, especially in TB which is also resistant to second-line therapies, and could also be applied to the design of effective vaccines and to better target treatment of latent TB. Evidence from both human and animal studies suggests that the most effective immune responses in TB comprise inflammation to control the bacteria balanced by regulatory mechanisms that prevent damage to the tissues ^3^, but the molecular details of how this balancing act is achieved are incompletely understood.

In order to elucidate the molecular mechanisms that lead to favorable outcomes, it is essential to faithfully model immune responses at the site of TB disease, more specifically within the granuloma, where key interactions between host and pathogen take place. Advances in genome editing techniques now provide unprecedented opportunities to investigate the influence of identified candidate genes or pathways by genetic manipulation in tractable animal models. Ultimately, these strategies are expected to identify pathways that represent novel therapeutic targets or biomarkers that could be applied to improve diagnosis, design effective personalized treatment plans and monitor clinical response to therapy.

Whilst eagerly awaiting new interventions emerging from a better understanding of pathogenesis, we also need to address the issues of inequality, human rights and social protection that underpin the TB, and DR-TB, epidemic. DR-TB disproportionately effects underserved populations, for example those of lower-socioeconomic status, people living with HIV, those living further away from health facilities and those who inject drugs. These factors strongly contribute to the burden of undiagnosed DR-TB and the inability to successfully complete treatment, leading to individual morbidity and mortality as well as ongoing transmission. A better understanding of how health systems, social and financial interventions can support DR-TB prevention and treatment is desperately needed.

## Hope for the future

In 2018 we have some hope for the future. In the UK we are moving into an era where whole-genome sequencing is used in clinical practice to rapidly assess the drug sensitivity of mycobacteria and provide a highly detailed picture of transmission. The Nix-TB regimen, which combines pretomanid, bedaquiline and linezolid (ClinicalTrials.gov Identifier: NCT02333799) offers the possibility of an effective 6-month treatment for all forms of drug resistant TB ^4^. There will also be a United Nations General Assembly, high-level meeting on TB taking place in 2018. This will be the first-ever high-level meeting on TB and the presence and commitment of heads-of-state will be fundamental to stimulate and drive efforts to control TB.

The TB community can learn lessons from the HIV community in the unified way that they responded to HIV. The quote below could equally apply to our response to DR-TB. The future lies in a concerted and integrated response to this threat which is focused not just on the mycobacteria but also the patient, the economy and the health systems that carry the burden of dealing with DR-TB.

*“our most precious contribution may well be that at the time of the plague we did not flee; we did not hide; and we did not separate.”-* Jonathan Mann.
